# Conventional Feed-Grade or Slow-Release Coated Urea as Sources of Dietary Nitrogen for Fattening Lambs

**DOI:** 10.3390/ani13223465

**Published:** 2023-11-09

**Authors:** Cristina Saro, Miguel Alonso Degeneffe, Sonia Andrés, Javier Mateo, Irma Caro, Lorena López-Ferreras, Egon Henrique Horst, Secundino López, Francisco Javier Giráldez

**Affiliations:** 1 Instituto de Ganadería de Montaña, CSIC-Universidad de León, Finca Marzanas s/n, Grulleros, 24346 León, Spain; miguelpuntoalonso@gmail.com (M.A.D.); sonia.andres@eae.csic.es (S.A.); s.lopez@unileon.es (S.L.); j.giraldez@eae.csic.es (F.J.G.); 2 Departamento de Producción Animal, Universidad de León, Campus Vegazana s/n, 24071 León, Spain; 3 Departamento de Higiene y Tecnología de los Alimentos, Universidad de León, Campus Vegazana s/n, 24071 León, Spain; jmato@unileon.es (J.M.); irma.caro@uva.es (I.C.); 4 Departamento de Pediatría e Inmunología, Obstetricia y Ginecología, Nutrición y Bromatología, Psiquiatría e Historia de la Ciencia, Universidad de Valladolid, Avda. Ramón y Cajal 7, 47003 Valladolid, Spain; 5 Departamento de Biología Molecular, Instituto de Biomedicina (IBIOMED), Universidad de León, Campus Vegazana, s/n, 24071 León, Spain; lorena.lopez.ferreras@unileon.es; 6 Department of Veterinary Medicine, Parana Midwestern State University, Guarapuava 85040-167, PR, Brazil; egonhh@yahoo.com.br

**Keywords:** Assaf, type of urea, feed efficiency, fermentation, carcass, meat

## Abstract

**Simple Summary:**

Reducing the environmental impact and the use of plant foods in animal feeding are current challenges of ruminant production systems. The use of non-protein nitrogen, especially urea, allows reducing the use of protein supplements of vegetable origin, which contributes to reducing both the competition for nutritional resources between humans and other animal species and the water and carbon footprints. This article provides new knowledge, comparing feed-grade conventional urea and slow-release urea under the conditions of intensive fattening of lambs, characterized by use of compound feed with a high content of starch-rich feeds. Our results suggest that replacing conventional feed-grade urea with slow-release urea in the diet of Assaf fattening lambs does not improve the feed efficiency, metabolic profile, or carcass or meat characteristics, and increases the feeding costs.

**Abstract:**

Twenty-two Assaf male lambs (29.2 ± 0.9 kg live weight and 89 ± 0.2 days of age), distributed in two experimental groups, were used to evaluate the use of either feed-grade conventional urea (Control diet; *n* = 11) or slow-release urea (SRU diet; *n* = 11) as sources of dietary nitrogen on animal performance, ruminal fermentation, blood acid-base status, plasmatic metabolic profile, and carcass and meat quality. Animals were housed individually and fed ad libitum. At the end of the fattening period (day 70), the animals were slaughtered to compare the fermentation patterns in ruminal digesta and to evaluate the carcass and meat characteristics. No statistically significant differences (*p* > 0.05) were observed between treatments in the dry matter intake, final live weight, average daily gain, and feed conversion rate. Regarding the ruminal fermentation parameters, the molar proportion of propionic acid was higher (*p* < 0.05) and that of butyric acid was lower (*p* < 0.05) with the SRU than with the Control diet. There were no significant differences (*p* > 0.05) between experimental treatments in the blood acid-base status and biochemical profile, except for the concentration of urea in plasma, which was significantly (*p* < 0. 05) greater in SRU than in Control lambs. No statistically significant differences were observed between treatments (*p* > 0.05) in the carcass and meat characteristics. In conclusion, the use of slow-release urea as a replacement for feed-grade conventional urea in the diet of Assaf fattening lambs, under the experimental conditions of this study, did not improve animal performance and increased the feeding costs.

## 1. Introduction

Non-protein nitrogen (NPN), especially urea, has been a widely used resource in ruminant feeding, targeting mainly an economic objective, since it allows reducing the use of protein supplements of vegetable origin, which are generally more expensive [1,2,3,4]. Its use can contribute to reducing the competition for nutritional resources between humans and other animal species and reduce both the water and carbon footprints [5,6]. For these reasons, this nutritional strategy is gaining new momentum, which is reflected in the research that is being carried out to maximize its use. 

Simultaneously with the incorporation of urea in ration formulation, an effort has been made to develop technological procedures to reduce the degradation rate of conventional urea in the rumen and to achieve a better synchronization between energy and nitrogen availability for rumen microorganisms [7,8].

Theoretically, the higher cost of SRU would be offset by increasing the efficiency of microbial protein synthesis and fiber digestibility and reducing ruminal ammonia and blood urea concentrations and N waste, which would result in a better balance of N and animal performance. However, there are not many in vivo studies comparing feed-grade urea, and the reported results are controversial. For instance, Mahmoudi-Abyane et al. [9] reported an increase in the relative population of fibrolytic bacteria, but also in the ruminal ammonia concentration, without effects on blood urea when replacing feed urea with SRU in fattening lambs. On the contrary, Alves et al. [10], also in fattening lambs, reported an increase in blood urea. Likewise, in beef steers, Taylor-Edwards et al. [11] observed that replacing feed-grade urea with SRU at different levels of dietary content (0.4, 0.9, 1.2, and 1.6% of urea) reduced both rumen ammonia and blood urea concentrations, but the body weight gain to feed ratio decreased at the lowest and the highest urea contents. Similarly, Tedeschi et al. [12] found a negative effect on body weight gain and feed conversion when replacing feed-grade urea with SRU in steers during the finishing phase. On the other hand, Bourg et al. [13], in growing cattle, and Savafi and Chaji [14], in fattening lambs, did not find differences in body weight gain and feed conversion when replacing feed-grade urea with different types of SRU. Furthermore, conclusive results have not been reported on the effects of the type of urea used as a dietary nitrogen supplement on carcass yield and fatness [12,15,16,17,18]. 

These controversial results suggest the intervention of dietary modulating factors, such as the feeding level, forage to concentrate ratio, fermentation rate of carbohydrates, content of urea, or type of coating method, among others. Consequently, the advantages of using SRU instead of feed-grade urea cannot be extrapolated to all conditions, and scientific knowledge is needed to establish recommendations for the efficient use of each type of urea. 

It is worth mentioning that most research evaluates partial aspects, and this limits comparisons between studies. Furthermore, to our knowledge, no studies have been carried out either in intensive fattening of lambs fed diets to achieve growth rates above 250 g/day nor to evaluate the effect of different types of urea on meat quality in fattening lambs. Taking this into account, the objective of this work was to study the effect of replacing urea with SRU on the animal performance, feeding costs, ruminal fermentation pattern, blood acid-base status, biochemical profile, and carcass and meat quality in heavy fattening of male Assaf lambs.

## 2. Materials and Methods

### 2.1. Animals and Diets

Twenty-two Assaf male lambs were distributed in two experimental groups, balanced for age and initial live body weight (on average, 29.2 ± 0.92 kg live weight and 89 ± 0.2 days of age). One of the groups (Control) received a total mixed ration (TMR) containing feed-grade conventional urea. The other group (SRU) received a similar ration, but feed-grade urea was replaced with SRU (Optigen^®^, Alltech Spain, Guadalajara, Spain). Experimental diets were formulated to be isoproteic and isoenergetic. The ingredients and chemical composition of the experimental diets are shown in Table 1. 

Lambs were housed in individual pens (1.45, 1.40, and 1.30 m in width, length, and height, respectively) during the whole experimental period (70 days) and fed the corresponding experimental diet ad libitum. Animals had free access to fresh water and were able to see and hear the other animals. Experimental conditions and handling practices followed the recommendations of the Directive 2010/63/EU of the European Parliament for the protection of animals used for experimental and other scientific purposes, as approved by the CSIC Animal Experimentation Committee and the Competent Authority (protocol number 624/2017).

### 2.2. Experimental Procedure

The experimental period lasted 70 days. Experimental diets were offered once a day in an appropriate amount to allow a minimum of 10% of refusals based on the previous day’s intake. Refusals were weighed daily, and samples were collected, pooled in weekly composites for each animal, and stored for subsequent analysis. 

Body weight was recorded on days 1, 14, 28, 42, 56, and 70 before the morning feeding. Blood samples were taken on days 1, 35, and 70 before morning feeding, and after having removed the refusals, to determine the biochemical profile. On day 66, blood samples were collected before (0 h) and 2 and 4 h after feeding 200 g of feed to study the postprandial variations in the biochemical profile. Blood samples were taken by jugular venipuncture into lithium heparin tubes, placed on ice, and then centrifuged at 3520× *g* for 20 min at 4 °C. Then, plasma samples were frozen at −80 °C until analysis. On days 35 and 70, an extra blood sample was taken and immediately transferred to the laboratory for determination of the acid-base status. 

### 2.3. Slaughter, Carcass, and Meat Quality Measurements

At the end of the experimental period, ram lambs were transferred to a commercial abattoir, where they were slaughtered 2 h after receipt. Lambs were stunned by electrocution, sacrificed by exsanguination, eviscerated, and skinned. The handling of the animals during transport and slaughter strictly followed Council Regulation (EC) No. 1099/2009 on the protection of animals at the time of slaughter.

Carcass weight was recorded immediately after slaughter (hot carcass weight, HCW). After refrigeration at 4 °C for 24 h, carcass was weighed again (CCW) and pH was measured on the *longissimus thoracis* muscle at the level of the 6th rib using a pH meter, equipped with a penetrating electrode and a temperature probe (Metrohm, Switzerland). Subcutaneous fat color parameters (L* (lightness), a* (redness), and b* (yellowness)) in the lumbar area were determined using a Minolta CM-2002 chroma meter (Konica-Minolta Sensing, Osaka, Japan), operating with the D65 illuminant, SCI mode, with a 10° visual angle, an 11 mm aperture for illumination, and an 8 mm aperture for measurement. 

Carcasses were carefully halved and both sides were weighed, and then the left-side carcass internal length (L), chest width (Th), and pelvic limb length (F) were measured, and the compactness index (ICC, CCW/carcass internal length) was calculated. Then, the left side was divided into commercial cuts (leg, foreribs, loin, shoulder, breast, neck, and tail), as described by Colomer-Rocher et al. [19], and these were weighed. Leg tissue composition was determined following the method of Fisher and de Boer [20].

The loin was transversally cut after the 13th rib and the subcutaneous fat depth and *L. lumborum* width and depth were measured with a caliper. Next, the *L. thoracis* and *L. lumborum* muscles were removed from the correspondent joints and weighed together. Then, two 2.5 cm-thick slices from the distal end of the *L. thoracis* muscle were cut and placed on a polypropylene tray, which was wrapped with polyvinylchloride cling film and stored in a refrigerator (4 °C) in darkness. At days 1 (2 h after storage) and 7 (6 days after storage), the tray was unwrapped, the slices were weighed, and the color was determined in duplicate on the upper surface of the slices following the procedure previously described for the color of subcutaneous fat. Moreover, in this case, the spectra were recorded and the reflectance ratio at wavelengths of 630 and 580 nm, indicating color changes in meat during storage [21], were calculated. A third 2.5 cm-thick slice was cut, packaged under vacuum, and cooked in water at 80 °C for 40 min; after cooling, it was immediately analyzed for thiobarbituric acid-reactive substances (TBARS) following the method of Nam and Ahn [22]. The remaining portion of *L. thoracis* was trimmed to eliminate connective tissue, minced in a food processor, and frozen at −20 °C until analysis. 

Cooking losses and meat hardness (shear force) were determined using the muscle *L. lumborum*, both at 24 h postmortem (day 1) and after 6 additional days (day 7) of refrigerated (4 °C) storage, as described by Santos et al. [23].

### 2.4. Ruminal Fermentation

In 4 representative lambs of each group, rumen was removed, and the content was mixed and straightened through 4 layers of cheesecloth. Then, pH was measured, 0.8 mL of fluid was added to 0.5 mL of deproteinizing solution (20 g/L of metaphosphoric acid and 0.6 g/L of crotonic acid) for volatile fatty acids (VFA) determination, and 4 mL was added to 20 µL of H_2_SO_4_ 20% (vol/vol) for ammonia-N analysis. 

The rest of the ruminal fluid was used for an in vitro trial to assess rumen fermentation by incubating the experimental diet that the animal had previously received. Strained ruminal fluid was stored in thermos flasks and immediately transported to the laboratory (from the slaughter to arrival at the laboratory, the process lasted around 1 h). Rumen fluid from each lamb was used as a separate inoculum and mixed with the culture medium described by Goering and Van Soest [24] in a 1:4 (*v*/*v*) proportion.

Incubations were performed in 120 mL serum bottles, in which 300 mg of dry matter (DM) substrate was weighed. Two bottles per substrate and animal and blanks were included in the incubation trial. Thirty milliliters of diluted rumen fluid was dispensed anaerobically in each bottle, which were immediately sealed with rubber stoppers and aluminum seals and placed in an incubator at 39 °C for 24 h. At the end of the incubation period, the total gas production was determined using a pressure transducer (Delta Ohm DTP704-2BGI, Herter Instrument SL, Barcelona, Spain) and a calibrated syringe, the bottles were swirled in ice to stop fermentation, and then they were opened to measure the pH in the incubation medium. Samples for ammonia-N and VFA analysis were taken as described for samples taken at slaughter.

### 2.5. Protein Degradability

A complementary in situ study was carried out to obtain additional information on the chemical and nutritional characteristics of the experimental diets and to verify that the type of urea modified the RDP content as expected. Three Assaf ewes, fitted with ruminal cannulae (40 mm inner diameter), as described by Dougherty [25], and fed the Control diet, were used. Nylon bags (125 × 100 mm in size and a 45 µm mesh size), containing 7 g of the corresponding diet (Control or SRU) ground to pass a 4 mm screen, were incubated in the rumen for 2, 6, 12, 24, and 48 h. Three bags (one per animal) were used for each time interval and diet. After incubation, the bags were removed, rinsed with tap water, and frozen for at least 5 days at −30 °C. Finally, all bags, including two extra bags per diet to estimate disappearance at zero time, were machine-rinsed using a cold-water program for 30 min. Dried bags were weighed, residues were ground to pass a 1 mm screen, and the CP content of each was determined.

### 2.6. Analytical Procedures

The DM contents of the feed offered, refusals, and residues of the in situ incubation were determined according to the ISO 6496:1999 procedure [26]. Ash, protein, and starch contents were analyzed following the procedures ISO 5984:2002 [27], ISO 5983:2009 [28], and ISO 6493:2000 [29], respectively. Crude fat was determined using the Ankom fiber bag technique [30], while NDF and ADF were determined, as described by Van Soest et al. [31], using the Ankom technique and expressed exclusive of residual ash.

VFA determination in the ruminal fluid samples was performed following the procedure described by Carro et al. [32]. The ammonia concentration was analyzed according to the colorimetric method proposed by Weatherburn [33].

Plasma samples were thawed overnight at 4 °C, and then the concentrations of aspartate aminotransferase (AST-GOT), alanine aminotransferase (ALT-GPT), urea, total protein, albumin, creatinine, triglycerides, total cholesterol, glucose, lactate, calcium, and phosphorus were analyzed using an automatic biochemical profile analyzer (ILAB 650, Instrumentation Laboratory, Lexington, MA, United States). To study the blood acid-base status, the pH, bicarbonate (HCO_3_^−^), anion gap, CO_2_ pressure (pCO_2_), and Na, K, and Cl concentrations were determined using a blood gas and electrolyte analyzer (VetStat, Idexx, Barcelona, Spain). The determination of the acid-base parameters was carried out immediately (within one hour) after the collection of blood samples.

Meat samples were freeze-dried to determine the dry matter content. Then, they were analyzed for ash (AOAC official method 920.153), CP (AOAC official method 981.10), and fat (AOAC official method 960.39) contents.

### 2.7. Calculations and Statistical Analysis

The values for the disappearance of crude protein (dg) with time were fitted to the model: $dg=a+b\times (1-e^{\left(-ct\right)})$ [34], using the NLIN procedure of the SAS package (SAS Inst. Inc., Cary, NC, USA). Effective rumen degradation (ED) of CP was calculated using the parameters *a*, *b*, and *c* and the rumen outflow rate (*kp*), according to the following equation:

$$ED=a+\frac{b\times c}{c+kp}$$

A kp value of 0.072 h^−1^ was used [35].

ADG (g/d) was estimated as the regression coefficient (slope) of body weight against time, and the feed conversion ratio was obtained by dividing the average daily dry matter intake (DMI) by the estimated ADG. 

Data on the feed intake, ADG, feed efficiency, in vivo and in vitro rumen fermentation parameters, carcass characteristics, and chemical composition of the meat were subjected to one-way analysis of variance, with the inclusion of diet as the fixed effect and the animal nested within the diet as the residual error. 

Data from the blood gases and biochemical parameters, color, cooking losses, and texture of the meat were analyzed as a repeated measures model, including in the model the fixed effects of the diet, the experimental day, and their interaction. The random effect of animals nested within the diet was used as the error to test the diet effect, and the mean square of the day × animal (diet) interaction was used as residual error to test the effects of day and the interaction between diet and day. Different covariance matrixes were evaluated based on Schwarz’s Bayesian information criteria. Plasma values at day 0 were used as covariates, being removed from the model when their effect was not significant (*p* > 0.05). 

All the analyses were performed using the MIXED procedure of SAS (SAS Inst. Inc., Cary, NC, USA). The level of significance was set at *p* < 0.05, and the least significant difference test was used for the multiple comparison of means. 

## 3. Results

### 3.1. Feed Intake, Animal Performance, and Ruminal Fermentation Parameters

There were no significant (*p* > 0.05) differences between experimental groups either in the feed intake or growth rate nor the feed to body weight gain ratio (Table 2).

In vivo and in vitro fermentation parameters are shown in Table 3. No differences (*p* > 0.05) were observed in ruminal pH, ammonia, and VFA concentrations measured in vivo, but lower (*p* > 0.05) proportions of acetate and butyrate and a higher (*p* > 0.05) molar proportion of propionate were observed in the rumen fluid of lambs fed the SRU compared with the control diet. Except for the acetate proportion (*p* > 0.05), similar effects on the fermentation parameters were found in the in vitro trial using experimental diets as a substrate.

### 3.2. Blood Acid-Base Status and Biochemical Profile

As shown in Table 4, none of the blood parameters related to acid-base status were significantly (*p* > 0.05) affected by the dietary treatments, although the HCO_3_^−^ concentration and pCO_2_ values decreased and the anion gap value increased with time in both experimental groups. Likewise, the type of urea had no effect (*p* > 0.05) on the plasma concentration of any of the measured parameters, except urea. Unexpectedly, the concentration of urea was significantly (*p* < 0.05) lower in Control- than in SRU-fed lambs. This effect was also observed when blood samples were taken at different hours after feeding (see Table 5). Urea was the only parameter affected by the type of diet (*p* < 0.05), with greater values for SRU lambs at any hour. Nevertheless, an interaction between diet and time was observed for ALT (*p* < 0.01), although it was a consequence of the differences within treatments and not between treatments. 

### 3.3. Carcass and Meat Characteristics

The effects of the type of urea on the carcass and meat characteristics are presented in Table 6 and Table 7. No differences were observed between dietary treatments in any of the parameters evaluated. Meat technological quality traits were affected by storage time, significantly (*p* < 0.05) increasing the cooking losses and colorimetric parameters (L*, a*, b*) and decreasing the hardness. The effect of storage time was independent of the diet consumed by the lambs (*p* > 0.05).

## 4. Discussion

### 4.1. Feed Intake, Growth Rate, Ruminal Fermentation, and Metabolic Blood Profile

The diets were formulated to allow the lambs to achieve their maximal potential growth and a low feed conversion ratio, according to previous studies carried out in heavy Assaf fattening lambs [36,37,38]. Moreover, experimental diets were designed to be isoproteic in terms of crude protein, but conventional feed-grade urea is more rapidly solubilized and degraded in the rumen than SRU and, thus, the calculated dietary rumen degradable protein (RDP) was greater and the rumen undegradable protein (RUDP) was lower in the Control diet than in the SRU diet. Some studies have reported that the substitution of conventional urea by SRU can reduce the ruminal ammonia concentration, theoretically favoring a better synchronization between fermentable energy release and protein degradation and improving the feed efficiency and animal performance [7,39,40,41,42]. However, in agreement with the results observed herein, these beneficial effects on animal performance are not always reported. For example, Mahmoudi-Abyane et al. [9] reported feed to gain ratios of 6.6 and 6.5 kg/kg in finishing lambs fed diets with feed-grade urea or SRU, respectively. Similar values were reported by Mayshayekhi et al. [17] in Arabian lambs. In the present study, better FCR values were observed, but the lambs received higher energy and protein diets and showed growth rates higher than those reported in the above-cited studies. 

Likewise, despite the greater RDP intake of lambs fed the control diet, no significant differences were observed in the ammonia concentration in the rumen, although mean values were numerically higher in control lambs. In our research, the urea content per kg is equivalent to 27 g of CP content (approximately 26% of the RDP) in both experimental diets, and this rather low proportion could limit the effect of the type of urea, especially in high-protein diets. Nevertheless, the lack of an effect of replacing conventional urea with SRU on the ruminal ammonia concentration has also been reported in vitro and in vivo when feeding diets with a lower CP content (8 to 15% on DM basis), where urea represented more than 25% of the total dietary CP [9,17,43,44,45]. The concentration of ammonia in the rumen is the result of the balance between the degradation and absorption of dietary or endogenous sources of N, and the synthesis of microbial protein. Therefore, a higher RDP intake is not always reflected in a higher concentration of ammonia in the rumen [36,44]. In fact, NH_3_ has buffer activity [46,47], and the lack of differences between dietary treatments in the ruminal pH and blood acid-base status would agree with the slight differences in the ruminal ammonia concentration. 

It has been reported that replacing urea with SRU rarely affects the ruminal metabolite concentration, except ammonia, at least in situations where ammonia does not limit microbial growth [11]. However, the fermentation pattern seems to differ between treatments, with a higher proportion of propionate and a lower proportion of butyrate in the animals of the SRU group. It should be noted that the in vivo data should be interpreted with caution, since they correspond to a single sampling (at the time of slaughter) and after a short period of fasting. However, the results obtained in the in vitro fermentation assays were consistent with those recorded in vivo.

It does not seem that the differences in fermentation can be attributed to a limited N supply, since the values recorded for both experimental groups were higher than those considered optimal for microbial growth [48]. The observed values were within the range reported by other authors in lambs with similar characteristics and consuming diets with a high proportion of concentrate [32,37,49,50].

The increase in the proportion of propionate caused by the substitution of conventional urea with SRU has been reported in previous studies and has been associated with an improved synchronization between protein degradation and carbohydrate fermentation [16,51]. 

However, in our study, no differences were observed in total VFA production, nor in feed efficiency, which would theoretically be expected from a more efficient microbial protein synthesis as a result of more synchronized energy and nitrogen release, and less ammonia absorption and energy expenditure for the synthesis of urea in the liver. 

Some studies have also evaluated the relationship between the plasmatic urea concentration and the efficiency of microbial protein synthesis in the rumen. Thus, for example, Valadares et al. [52] suggested that in cattle, a range of plasmatic urea concentration from 13.5 to 15.2 mg/dL corresponds to the maximum microbial efficiency, from which a maximal inefficiency in the use of nitrogen would occur, while Oliveira et al. [53] indicated that the limit from which greater losses of dietary N occur is 19–20 mg/dL. In the present study, urea concentrations in plasma were much higher than those indicated in the cited works, suggesting an excess of nitrogen in both treatments for the synthesis of microbial protein. Regarding the molar proportion of butyric acid, no effects [9,11,14,16,42,54,55], increases [17,56,57], or reductions [44] have been reported, but these changes have been related with an opposite change in the proportion of acetic acid. However, in the present study, the proportion of acetic acid decreased in the in vivo trial and was not affected in the in vitro assay. However, although no significant differences were observed in the ruminal ammonia concentration, this was 10% higher when feed-grade urea was used, and it has been reported that as ammonia increases, there is a shift from propionate to higher proportions of butyrate and acetate [58]. On the other hand, even without differences in the concentration of ammonia in the rumen, some authors have reported changes in the microbiota when replacing urea with SRU [9,45], which could be the reason for the differences observed in the fermentation pattern in our study.

It should be noted that, despite the differences observed in the fermentation pattern, except for the plasma concentration of urea, no other differences were observed between treatments in the metabolic profile, with the parameters within the range of values observed for animals with similar characteristics [37,38].

The plasma concentration of urea was higher in the animals of the SRU group. It should be noted that differences were observed on days 35 and 70, before feeding, and even on day 66 after 2 and 4 h of feeding, which is considered a consistent result. Similar differences have been observed in other studies [9,10], but it is difficult to explain the reasons for such effect. It is expected that in SRU lambs, a higher proportion of urea bypasses the rumen and is degraded to NH_3_ by the intestinal microbiota, being partially absorbed. This shift in the site of degradation and absorption could modify the entry rate of endogenous urea into the post-ruminal digestive tract, and even modify the urea/NH_3_ concentration ratio in plasma and urine. It is also possible that in control lambs, a fast and high absorption of ammonia may punctually exceed the ureagenesis capacity of the liver and result in increased conversion of ammonia to glutamine and in more excretion of ammonia in urine. In this regard, it has been reported that the urea-N/NH_3_-N ratio in sheep urine can vary between 0.7 and 24.5 depending on the feeding conditions [59]. On the other hand, as mentioned previously, the fermentation pattern was modified by the type of urea, increasing the proportion of butyrate in lambs fed with conventional urea. It has also been reported that butyrate increases both rumen epithelial protein synthesis and urea transfer from plasma to the rumen [60], which could cause a lowering of the plasma urea concentration. 

### 4.2. Carcass and Meat Quality and Feeding Costs 

The values obtained from carcass characteristics agreed with those reported in previous studies in fattening Assaf lambs [37,38,61] and were not altered by the type of urea used. Carcass weight and conformation are highly related to age and body weight at slaughter. These were not affected by dietary treatments since lambs of both treatments showed equal growth rates and were slaughtered at the same age. Similar results have been reported in beef cattle [12,13,18,61] and fattening lambs [17]. Alves et al. [10] found a quadratic effect of the proportion of conventional urea replaced with SRU on carcass yield in lambs, demonstrating a minimum effect when only 40% of conventional urea was replaced. However, no differences were expected when 100% of conventional urea was replaced, which would be consistent with our results. 

As expected [38,62,63], meat quality was modified during aerobic storage, increasing all colorimetric parameters (L*a*b*) and cooking losses and decreasing the cooked meat shearing force. This effect was independent of the type of urea used.

Replacing conventional urea with SRU did not modify any of the meat quality parameters evaluated. To our knowledge, there are not many studies focusing on the effect of the type of urea on meat quality. In concordance with our study, Bourg et al. [13] did not observe differences in the meat chemical composition when comparing conventional urea with SRU in finishing steers. Cardoso et al. [64] reported a reduction in the marbling score when using SRU instead of feed-grade urea, but this effect was not supported by differences in the meat chemical composition.

The lack of differences in meat technological parameters (cooking losses, shearing force, and colorimetric parameters) agreed with the lack of effect of the type of urea on the meat pH and chemical composition, with values within the range reported in the literature for heavy fattening Assaf lambs [37,65]. 

Feeding costs are generally the main component of production costs in livestock farms and, therefore, it is important to develop feeding strategies to reduce them. It has been reported that dietary supplementation of SRU improves the profitability of beef production when included in low-protein diets or replacing vegetable protein supplements [8]. However, SRU is more expensive than conventional feed-grade urea, which in the present study resulted in a 2.9% increase in feed costs compared to the Control diet. Consequently, from a strictly economic point of view, its use should also improve feed efficiency to be cost-effective when used instead of conventional urea. However, both the feed to gain ratio and the cold carcass yield were unaffected by the dietary treatments and, consequently, the cost of feeding expressed per kg of ADG (1.86 vs. 1.95 EUR/kg ADG for Control and SRU, respectively) or kg of CCW (1.48 vs. 1.52 EUR/kg CCW for Control and SRU, respectively) increased by 4.3 and 2.6%, respectively, suggesting that the use of SRU does not provide a relevant economic benefit.

## 5. Conclusions

Under the conditions of the trial reported herein, replacing conventional feed-grade urea with slow-release urea increased the blood urea concentration and did not improve animal performance or have any effect on carcass or meat characteristics. As the use of slow-release urea would have an evident impact on the cost of the diet, the inclusion of protected urea in fattening lamb diets seems of low practicability. Furthermore, some coated procedures to slow urea degradation in the rumen, such as that used in this study, could hamper ration pelleting, which is an emerging practice in intensive lamb finishing systems. Nevertheless, more studies are necessary to evaluate the effects with diets of different natures and understand the interaction between the types of urea, ruminal microbiome, fermentation patterns, and the molecular mechanisms involved in the urea transport processes through the ruminal wall.

## Figures and Tables

**Table 1:** Ingredients, nutritive value, and costs of the experimental diets (Control: formulated with feed-grade urea; SRU: formulated with slow-release urea).

	Control	SRU
**Ingredients, g/kg**		
Barley straw	150.0	150.0
Barley	490.5	490.5
Corn	189.0	190.0
Soybean meal	115.0	115.0
Molasses	10.0	10.0
Feed-grade urea	9.5	-
Slow-release coated urea ^1^	-	10.5
Soybean oil	6.0	4.0
Vitamin mineral premix	25.0	25.0
Sodium bicarbonate	5.0	5.0
**Composition, g/kg dry matter (DM)**		
DM, g/kg	881	886
Neutral detergent fiber (NDF) ^2^	242	223
Acid detergent fiber (ADF) ^1^	72	69
Crude protein (CP)	177	179
Rumen degradable protein (RDP) ^3^	115	101
Rumen undegradable protein (RUDP) ^4^ Crude fat	62 30	78 31
Starch	406	410
Ash	78	64
Metabolizable energy, Mcal/kg DM	2.80	2.79
Cost, EUR/kg DM	0.379	0.392

^1^ Optigen^®^; ^2^ Expressed without residual ash; ^3^ RDP = CP × effective degradability estimated at a rate of passage of 0.072 h^−1^; ^4^ RUDP = CP – RDP.

**Table 2:** Mean values of feed intake, average daily gain, and feed efficiency of heavy Assaf lambs fed diets with either conventional urea (Control) or a slow-release urea (SRU).

	Control	SRU	SED ^1^	*p*-Value
Dry matter intake, g/day	1440	1350	62.4	0.17
Crude protein intake, g/day	259	246	10.7	0.26
Average daily gain, g/day	295	277	16.2	0.27
Final body weight, kg	49.6	48.1	1.72	0.54
Feed conversion ratio, g/g	4.92	4.91	0.193	0.80

^1^ SED: standard error of the difference.

**Table 3:** Mean values of pH and volatile fatty acids (VFA) and ammonia concentrations in the rumen fluid, and in vitro fermentation parameters (gas and VFA production and ammonia concentration using experimental diets, starch, and NDF-straw as substrates) of heavy Assaf lambs fed diets with either conventional urea (Control) or a slow-release urea (SRU).

	Control	SRU	SED ^1^	*p*-Value
**In vivo parameters**				
pH	6.18	6.22	0.566	0.9236
Ammonia-N, mg/L	120	109	55.54	0.7814
VFA concentration, mmol/L	165	133	53.07	0.4369
Acetate, %	57.8	51.3	2.60	0.0124
Propionate, %	18.8	36.5	3.58	0.0004
Butyrate, %	18.5	7.3	4.39	0.0111
Branched fatty acids, %	3.18	2.66	1.237	0.5741
Valerate + caproate, %	1.76	2.23	0.859	0.4709
Acetate/propionate	3.13	1.42	0.383	0.0007
**In vitro fermentation using experimental diets as a substrate**				
pH	6.49	6.66	0.097	0.1163
Gas production, mmol	1.87	1.64	0.106	0.0711
Ammonia-N, mg/L	292	246	36.41	0.2428
VFA production, mmol	3.38	3.08	0.299	0.3455
Acetate, %	47.0	49.0	3.95	0.6294
Propionate, %	29.3	37.5	2.72	0.0236
Butyrate, %	20.0	7.93	3.39	0.0121
Branched fatty acids, %	1.88	2.78	0.727	0.1306
Valerate + caproate, %	1.88	2.76	0.483	0.1181
Acetate/propionate	1.63	1.33	0.233	0.2385

^1^ SED: standard error of the difference.

**Table 4:** Blood acid-base status and biochemical profile of heavy Assaf lambs fed diets with either conventional urea (Control) or a slow-release urea (SRU).

	Dietary Treatment		SED ^1^	Sampling Day		SED ^2^	*p*-Value		
	Control	SRU		35	70		Diet	Time	Diet × Time
**Acid-base status**									
pH	7.42	7.43	0.017	7.42	7.43	0.013	0.5459	0.5681	0.6141
pCO_3_, mm Hg	44.6	44.2	1.75	46.3	42.5	1.68	0.8175	0.0359	0.9786
HCO_3_¯, mmol/L	26.9	27.2	0.53	27.9	26.1	0.294	0.5486	0.0001	0.1497
Anion gap, mmol/L	15.4	15.4	0.67	14.4	16.4	0.672	0.9681	0.0087	0.3165
tCO_2_, mmol/L	28.2	28.7	0.50	29.5	27.4	0.35	0.3971	0.0001	0.1011
Na, mmol/L	148	148	0.53	149	147	0.5	0.2450	0.0057	0.8470
K, mmol/K	5.55	5.38	0.233	5.75	5.18	0.154	0.4561	0.0014	0.5048
Cl, mmol/L	111	111	0.67	112	110	0.7	0.9465	0.0096	0.8361
**Biochemical profile**									
Urea, mg/dL	37.88	48.42	1.605	40.69	45.61	1.604	0.0001	0.0066	0.2503
Protein, g/L	62.90	62.69	1.557	60.17	65.43	1.557	0.8920	0.0001	0.6922
Albumin, g/L	37.31	37.77	0.862	36.60	38.47	0.631	0.5980	0.0077	0.1532
ALT, U/L	18.21	17.44	1.168	17.51	18.15	0.544	0.5176	0.2586	0.5242
AST, U/L	93.02	94.78	6.008	90.55	96.95	3.065	0.8110	0.0499	0.3371
Creatinine, mg/dL	1.07	1.05	0.034	1.03	1.09	0.014	0.7850	0.0004	0.4117
Glucose, mg/dL	110	105	2.698	109	106	2.248	0.0740	0.2742	0.9443
LD, mg/dL	18.97	20.14	3.841	20.85	18.27	2.345	0.7641	0.2860	0.1961
Cholesterol, mg/dL	70.88	74.63	3.901	73.14	74.63	3.901	0.3481	0.8088	0.3785
Triglycerides, mmol/L	46.67	43.19	3.863	45.58	44.29	2.578	0.3790	0.6226	0.1314
Ca, mg/dL	11.57	11.57	0.190	11.45	11.70	3.130	0.9998	0.1271	0.0317
P, mg/dL	8.49	8.51	0.353	8.72	8.27	0.289	0.9564	0.1357	0.7403

^1^ SED: standard error of the difference comparing dietary treatments. ^2^ SED: standard error of the difference comparing days.

**Table 5:** Biochemical profile of heavy Assaf lambs fed diets with either conventional urea (Control) or a slow-release urea (SRU) assessed 0, 2, and 4 h post-feeding.

	Dietary Treatment	Time after Feeding			SED ^1^	SED ^2^	*p*-Value		
		0 h	2 h	4 h			Diet	Time	Diet × Time
Urea, mg/dL	Control	42.5 ^a^	46.7 ^b^	44.3 ^a^	2.342	0.711	0.0140	0.0051	0.0053
	SRU	51.6 ^c^	51.5 ^c^	49.3 ^b^					
Protein, g/L	Control	64.6	62.3	62.8	2.043	0.303	0.8801	0.0001	0.7422
	SRU	65.2	62.5	63.0					
Albumin, g/L	Control	38.0	36.7	37.4	1.297	1.369	0.0570	0.6684	0.3595
	SRU	39.8	41.6	38.6					
ALT, U/L	Control	16.3 ^b^	15.0 ^a^	15.0 ^a^	1.275	0.416	0.7622	0.7245	0.0440
	SRU	16.6 ^b^	15.9 ^ab^	15.0 ^a^					
AST, U/L	Control	91.3	86.9	89.7	7.527	1.263	0.4319	0.0003	0.5864
	SRU	98.7	91.8	95.5					
Creatinine, mg/dL	Control	1.09	1.07	1.08	0.038	0.008	0.8591	0.0014	0.3790
	SRU	1.10	1.06	1.05					
Glucose, mg/dL	Control	110	108	105	4.166	0.953	0.2114	0.0493	0.6101
	SRU	105	103	105					
LD, mg/dL	Control	18.6	14.1	13.4	3.208	1.975	0.8284	0.0451	0.5830
	SRU	18.0	17.2	13.0					
Cholesterol, mg/dL	Control	70.2	67.1	67.4	4.927	0.576	0.7801	0.0002	0.3873
	SRU	70.7	68.8	69.4					
Triglycerides, mmol/L	Control	44.1 ^b^	39.2 ^b^	27.2 ^b^	2.525	1.875	0.3389	0.0001	0.2070
	SRU	44.2 ^b^	39.4 ^b^	26.9 ^a^					
Ca, mg/dL	Control	12.0	11.6	11.9	0.172	0.146	0.8976	0.0005	0.2686
	SRU	12.3	11.5	11.8					
P, mg/dL	Control	8.83	8.44	7.86	0.537	0.172	0.6875	0.0001	0.2796
	SRU	8.93	8.03	7.50					

^1^ SED: standard error of the difference comparing dietary treatments. ^2^ SED: standard error of the difference comparing sampling times. ^a,b,c^ Means with superscripts are significantly different (*p* < 0.05).

**Table 6:** Carcass characteristics of heavy Assaf lambs fed diets with either conventional urea (Control) or a slow-release urea (SRU).

	Control	SRU	SED ^1^	*p*-Value
Cold carcass weight, kg	25.6	24.2	1.28	0.2956
Dressing percentage, %	51.7	50.4	0.660	0.0694
Chilling losses, %	1.03	0.99	0.025	0.1207
pH, 24 h	5.75	5.77	0.06	0.6819
Pelvic and renal fat, %	326	267	60.3	0.3314
Proportion of cuts ^2^, %				
Higher-priced joints	61.1	60.3	0.4	0.4018
Medium-priced joints	18.7	18.6	0.34	0.8289
Lower-priced joints	20.2	21.1	0.43	0.0913
Morphological parameters				
L, cm	65.6	65.5	1.12	0.9173
F, cm	42.6	42.3	0.60	0.6254
TH, cm	28.2	28.2	0.46	0.9378
ICC, g/cm	292	280	14.4	0.4192
Subcutaneous fat color				
L*	68.0	66.8	1.70	0.5072
a*	2.76	3.00	0.52	0.6639
b*	8.17	7.86	0.79	0.6941
Leg tissue composition, %				
Muscle	60.0	59.3	1.208	0.5573
Fat	17.7	16.9	1.209	0.5405
Bone	20.8	21.8	0.353	0.0596
Others	1.49	1.98	0.250	0.0667
Loin rib characteristics				
Weight, g	602	570	27.6	0.2627
Area, cm^2^	58.6	54.5	4.28	0.3551
Fat over rib, cm	0.89	0.96	0.132	0.5930

^1^ SED: standard error of the difference. ^2^ Legs, loin, and foreribs comprised the higher-priced joints, shoulders comprised the medium-priced joints, and the lower-priced joints included breast, neck, and tail.

**Table 7:** *Longissimus thoracis* composition, thiobarbituric acid-reactive substances (TBARS), and *Longissimus lumborum* cooking losses, texture, and color in heavy Assaf lambs fed diets with either conventional urea (Control) or a slow-release urea (SRU).

	Dietary Treatment		SED ^1^	Storage Day		SED ^2^	*p*-Value		
	Control	SRU		1	7		Diet	Time	Diet × Time
Chemical composition (g/kg)									
Water	75.5	76.6	0.80	-	-	-	0.2154	-	-
Crude protein	16.2	14.5	1.77	-	-	-	0.1778	-	-
Crude fat	2.32	2.51	0.189	-	-	-	0.3271	-	-
Ash	0.98	0.91	0.080	-	-	-	0.3671	-	-
TBARS (µg MDA ^3^/g sample)	3.23	3.05	0.270	-	-	-	0.5015	-	-
Cooking losses, %	21.2	20.5	1.58	19.5	22.2	0.96	0.6925	0.0121	0.8282
Texture, shearing force, N	83.0	85.4	6.25	92.5	76.0	2.97	0.7041	<0.0001	0.2367
L*	38.2	38.2	0.71	37.7	39.2	0.46	0.5770	0.0036	0.6459
a*	8.26	8.58	0.323	7.71	9.14	0.222	0.3310	<0.0001	0.0465
b*	9.61	9.71	0.479	8.44	10.88	0.453	0.8392	<0.0001	0.3637
630/580 ^4^	2.63	2.72	0.093	3.03	2.32	0.083	0.3338	<0.0001	0.1313

^1^ SED: standard error of the difference comparing dietary treatments. ^2^ SED: standard error of the difference comparing days. ^3^ MDA: malondialdehyde. ^4^ Ratio of the reflectance at wavelengths of 630 and 580.

## Data Availability

The datasets used and/or analyzed during the current study are available from the corresponding author upon reasonable request.
